# Morphometric and Anatomical Analysis of the Deltoid Ligament Complex: A Cadaveric Study in the Southeast Asian Population

**DOI:** 10.7759/cureus.81442

**Published:** 2025-03-29

**Authors:** Hui-Shan Angela Lim, Jun Rui Don Koh, Socklingam Raj Kumar, Ing How Moo, Kam King Charles Kon

**Affiliations:** 1 Orthopaedic Surgery, Changi General Hospital, Singapore, SGP

**Keywords:** anatomy, ankle, deltoid ligament, instability, morphology

## Abstract

Introduction

The deltoid ligament complex (DLC) confers stability of the ankle joint. This study quantitatively analyzes the morphometry of the DLC in the Southeast Asian population, highlighting anatomical variations relevant to surgical procedures.

Methodology

Seven embalmed amputated limbs were dissected. The width and length of the superficial and deep layers with their corresponding bands were measured using a metric ruler.

Results

The mean width of the superficial layer of the DLC was 20.43 + 2.72 mm at the origin, 20.00 + 1.07 mm at midway, and 15.29 + 1.28 mm at insertion. The tibionavicular ligament (TNL), tibiocalcaneal ligament (TCL), deep posterior tibiotalar ligament (dPTTL), and the deep anterior tibiotalar ligament (dATTL) were present in all specimens. In the superficial layer, the mean length of the TNL and TCL was 38.57 + 3.58 mm and 34.29 + 6.47 mm, respectively. In the deep layer, the mean length of the dPTTL and dATTL was 18.71 + 1.83 mm and 20.29 + 2.31 mm, respectively.

Conclusion

While the prevalence of the components of the DLC varies widely across the literature, it was present in all specimens of our study. The longest and shortest bands of the DLC were the TNL and dPTTL, respectively, concurring with current literature. However, the mean length of TCL, dPTTL, and dATTL in the Southeast Asian population appeared to be longer than that reported in a meta-analysis of European cadaveric studies. Knowledge of the morphology and anatomical variations of each component of the DLC in the Southeast Asian population is crucial to improve surgical management of medial ankle instability.

## Introduction

The deltoid ligament complex (DLC) is crucial for medial ankle stability. It primarily restricts anterior, posterior, and lateral translation of the talus while preventing excessive abduction [1,2]. Among all ankle sprains, 20% to 40% result in chronic instability [3] and 18% involve the DLC [4]. However, isolated DLC injury is rare [5] and is generally accompanied by concurrent lateral ankle ligament injury [6] or fracture of the medial malleolus [7]. Surgery is usually recommended if conservative treatment fails or if patients report chronic instability affecting their daily activities [8]. With advancements in anatomical reconstruction or repair of the ligament via open or arthroscopic surgery [9], the morphology of the DLC needs to be fully understood for successful procedures [10,11].

The DLC is a strong triangular band attached by its apex to the anterior and posterior borders and the tip of the medial malleolus [12]. It is composed of two layers, the superficial and deep layer [13]. Based on the Milner and Soames classification [14], the superficial layer comprises two major components: the tibiospring ligament (TSL) and the tibionavicular ligament (TNL), and two additional bands - the superficial posterior tibiotalar ligament (sPTTL) and the tibiocalcaneal ligament (TCL). The deep layer comprises a major component, namely the deep posterior tibiotalar ligament (dPTTL), and an additional band called the deep anterior tibiotalar ligament (dATTL). However, the prevalence and size of the various components have been inconsistently reported in current literature [10,14-20].

Additionally, while there is a meta-analysis done by Yammine et al. [21], it analyzed eight cadaveric studies comprising 142 ankles in total; all eight cadaveric studies comprised only European cadavers from the North American and British population. To our knowledge, only one study was done in Korea that analyzed 60 ankles from 39 cadavers. A study by Zhang et al. reports differences in anterior cruciate ligamentous anatomy between Chinese and Caucasians [22]. Therefore, our paper aims to identify and analyze the variations in morphology of the DLC in a Southeast Asian population to supplement further understanding of the anatomy for proper surgical reconstruction to achieve ankle stability.

## Materials and methods

A descriptive study was performed utilizing seven embalmed limbs (six right; one left) without background information, including age, sex, and demographic data. Limb specimens with visible necrosis, surgical scars, or gross anatomical deformities in the medial malleolus or surrounding structures were excluded from the study.

The skin and subcutaneous tissue were carefully dissected to expose the superficial layer of the DLC, the great saphenous vein, tibialis posterior tendon, and the rest of the tendon sheaths. The components of the superficial layer were identified, and the width (anterosuperior to posteroinferior distance) at the origin, midpoint, and insertion sites were obtained. Further dissection of the tibialis posterior tendon and flexor digitorum longus was done to measure the length superficial components: TCL and TNL. Thereafter, the superficial layer was removed to observe and measure the length of the deep layer components - dATTL and dPTTL.

The entire procedure was completed in a day including the dissection, analysis and measurements. The measurements were taken with a one-foot professional millimeter ruler. After tabulation of results, data analysis was performed using SPSS Statistics version 29.0 (IBM Corp. Armonk, NY, USA). The data was presented as mean ± standard deviation (SD).

## Results

**Figure 1:**
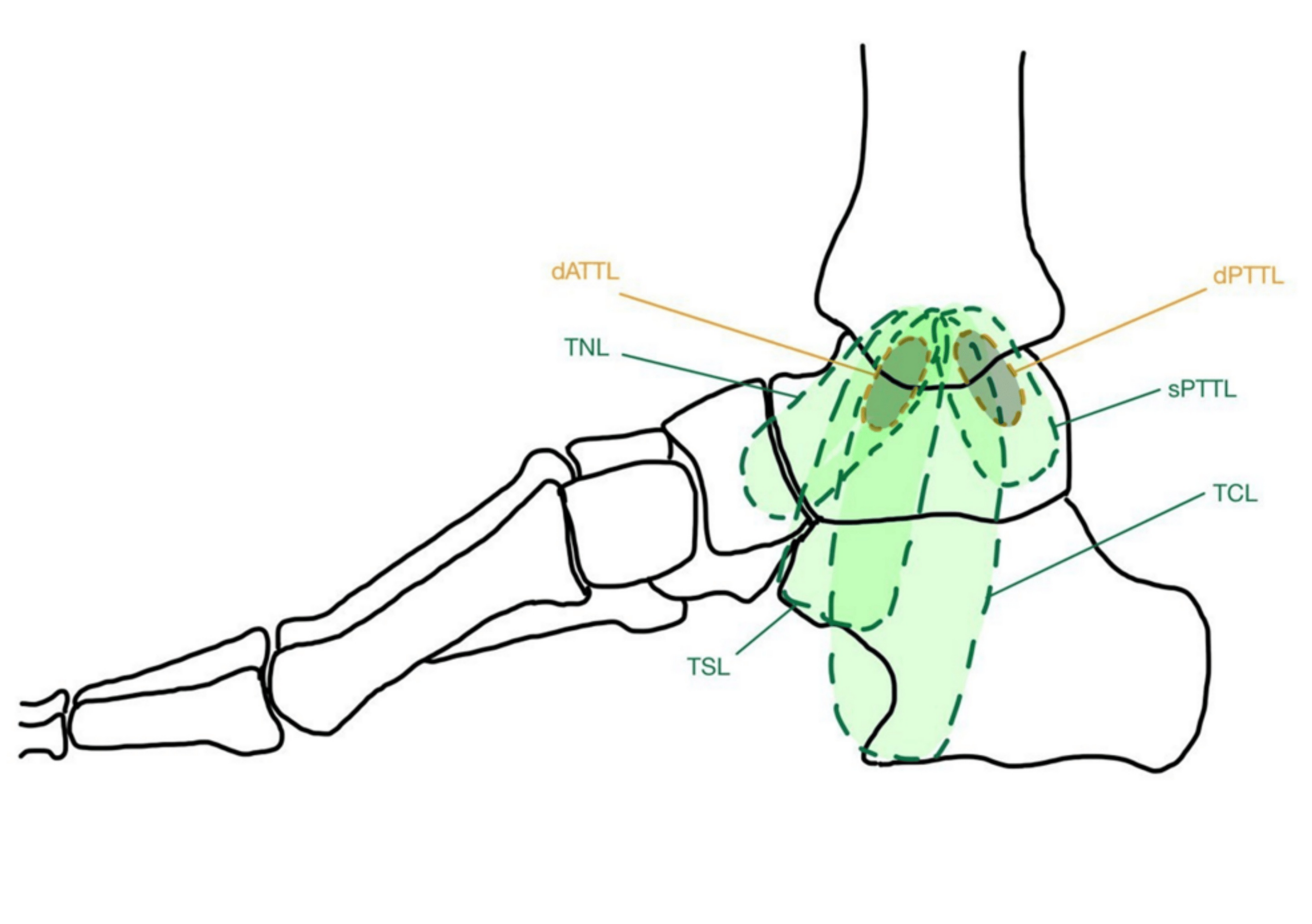
Medial view diagram showing both superficial and deep layer of the DLC Diagram created by the authors using the following reference: "Morphometric Study and Anatomical Variations of the Medial Ligament of the Talocrural Joint," by Sepúlveda R et al. [23]. DLC: Deltoid ligament complex, dATTL: Deep anterior tibiotalar ligament, dPTTL: Deep posterior tibiotalar ligament, TNL: Tibionavicular ligament, sPTTL: Superficial posterior tibiotalar ligament, TCL: Tibiocalcaneal ligament, TSL: Tibiospring ligament

The DLC macroscopically comprises a superficial layer and a deep layer with different ligamentous attachments (Figure 1) [22]. The respective measurements of the different components of the layers are summarized in Table 1.

**Table 1:** Summarized results of superficial and deep layer measurements TNL: Tibionavicular ligament, TCL: Tibiocalcaneal ligament, dPTTL: Deep posterior tibiotalar ligament, dATTL: Deep anterior tibiotalar ligament

Layer		Mean (mm)	SD
Superficial layer	Width at origin	20.43	2.72
	Width at midway	20.00	1.07
	Width at insertion	15.29	1.28
	Length of TNL	38.57	3.58
	Length of TCL	34.29	6.47
Deep layer	Length of dPTTL	18.71	1.83
	Length of dATTL	20.29	2.31

Superficial layer

**Figure 2:**
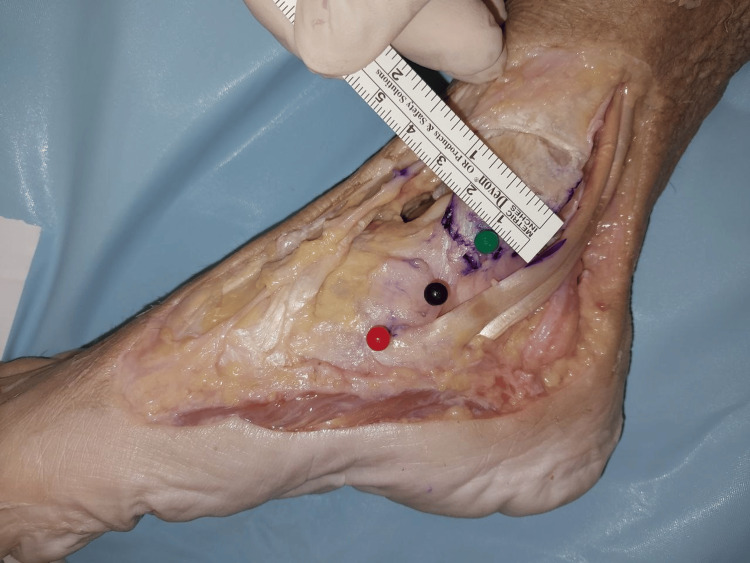
Width of superficial layer at origin

**Figure 3:**
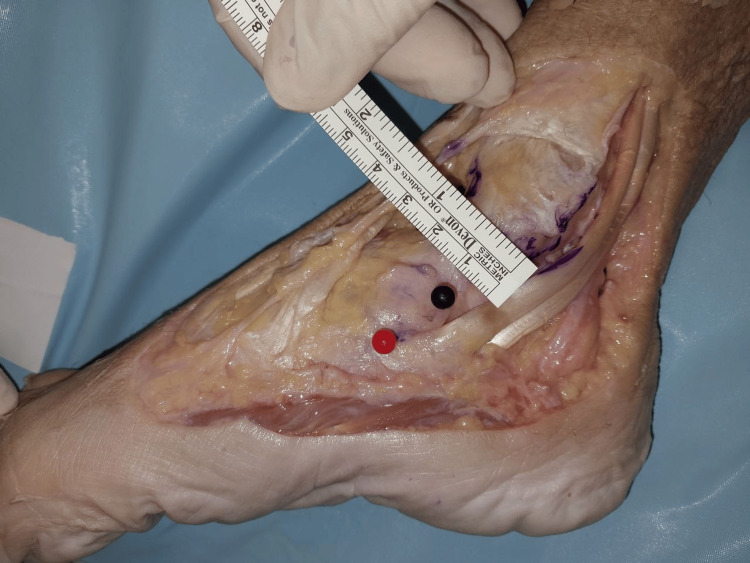
Width of superficial layer at midway

**Figure 4:**
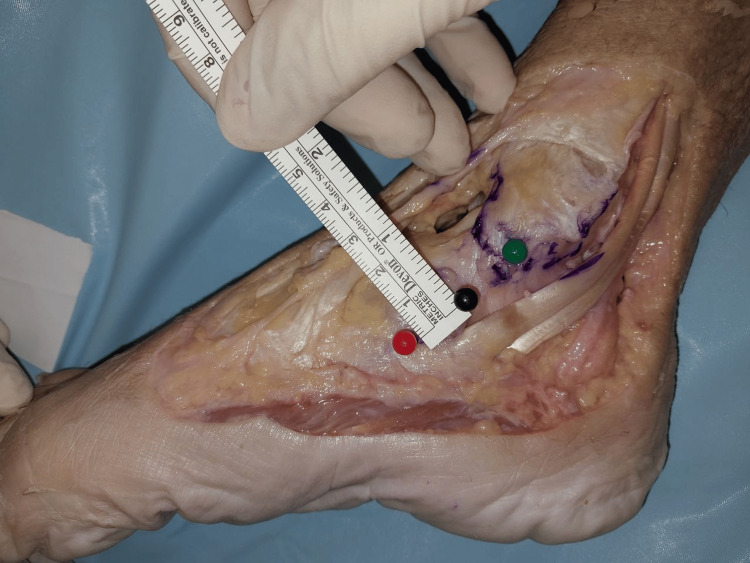
Width of superficial layer at insertion

The width of the superficial layer was measured at the origin, midway, and at the point of insertion (Figures 2-4). The mean width of the superficial layer of the DLC was 20.43 + 2.72mm at the origin, 20.00 + 1.07mm at midway, and 15.29 + 1.28mm at insertion.

**Figure 5:**
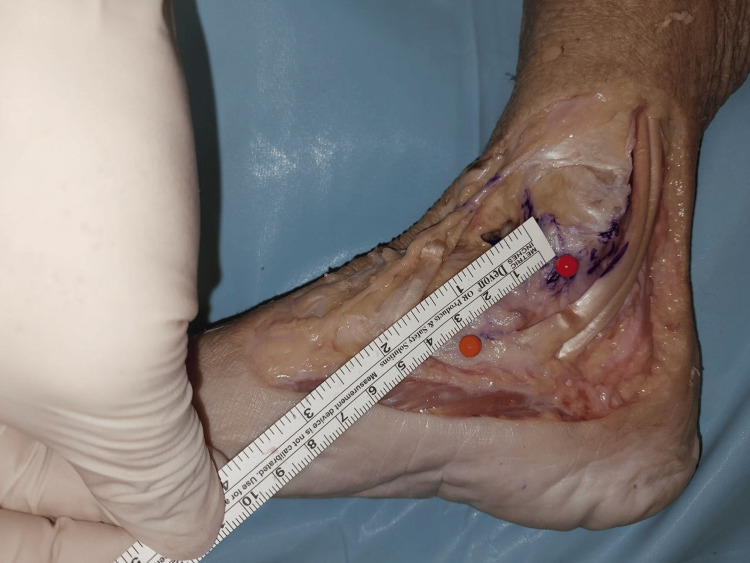
Length of TNL TNL: Tibionavicular ligament

**Figure 6:**
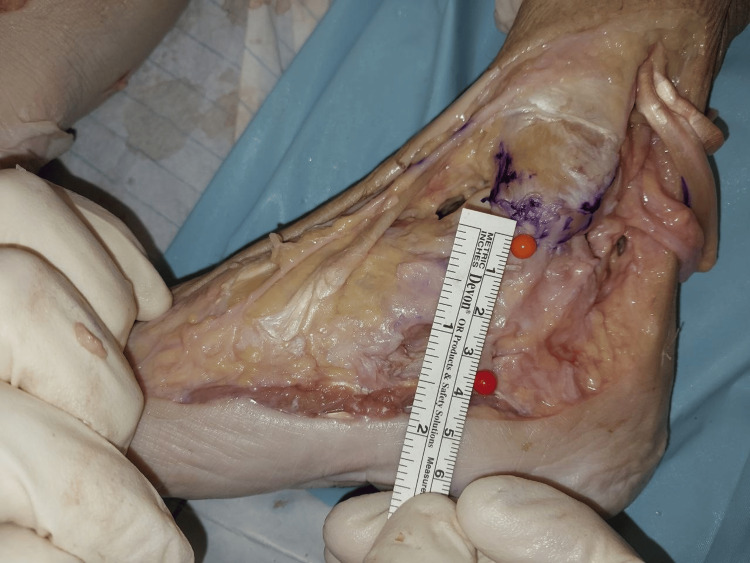
Length of TCL TCL: Tibiocalcaneal ligament

The length of the two major components of the superficial layer consists of the TNL with a mean length of 38.57 + 3.58 mm (Figure 5), and TCL with a mean length of 34.29 + 6.47mm (Figure 6).

Deep layer

**Figure 7:**
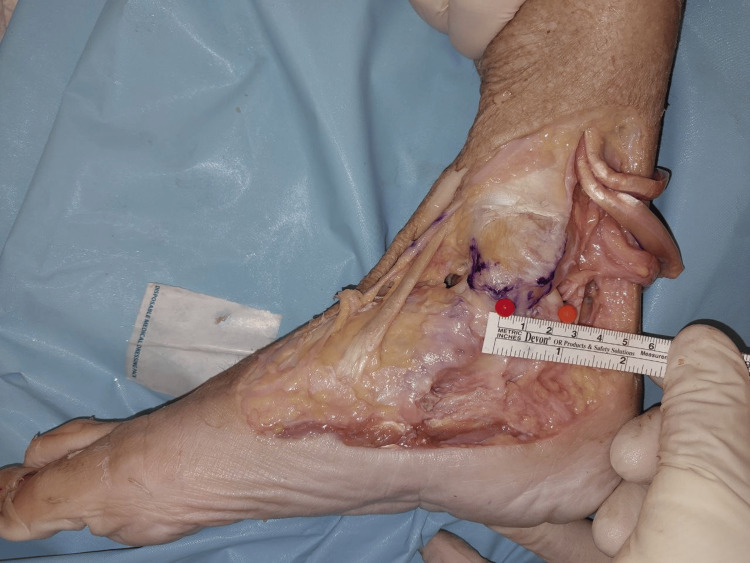
Length of dPTTL dPTTL: Deep posterior tibiotalar ligament

**Figure 8:**
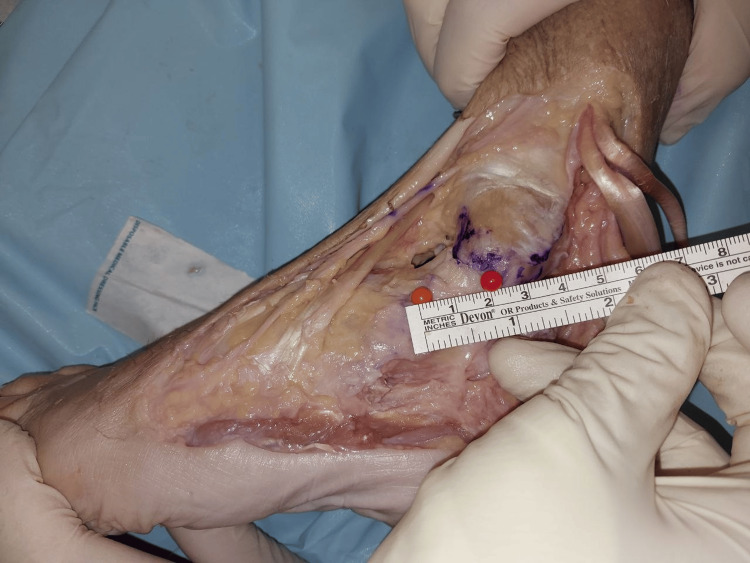
Length of dATTL dATTL: Deep anterior tibiotalar ligament

The major component of the deep layer, i.e., the dPTTL, had a mean length of 18.71 + 1.83mm (Figure 7), while the additional dATTL band had a mean length of 20.29 + 2.31mm (Figure 8).

## Discussion

Injury of the DLC often requires surgery, especially in patients with large or complete tears that fail to heal primarily with the development of chronic medial ankle instability [10,20] and early tibiotalar osteoarthritis in the long run [24]. Around 83% of lateral ankle ligament injuries tend to have concurrent DLC involvement [15]. Patients with posterior tibial tendon rupture or insufficiency and subsequent pes planus deformity can also precipitate failure of the DLC due to increased strain [25], and reconstruction of the posterior tibial tendon would require surgical correction of the DLC and spring ligament complexes [25-28]. Additionally, in total ankle arthroplasty, it is crucial to assess the integrity of the DLC to achieve good ligamentous balance [29] and good outcomes.

A recent study by Loozen et al., after careful review of the literature, recommends full deltoid repair in patients, whether arthroscopically or with open suture tape augmentation, even in the acute setting [9]. While some authors recommend combined TCL and dATTL repair [30], and some recommend dATTL repair only [31], Loozen et al. recommend the repair of both the dPTTL and dATTL to provide better stability in the axial and coronal plane [9]. Furthermore, in the setting of planovalgus foot with subtalar dislocation, further assessment and treatment of the TCL and TSL were recommended [32,33]. Therefore, as surgical repair of the DLC gains traction, proper understanding of the anatomy and morphology of the DLC is critical to aid in further advancements in anatomical repair and reconstruction guidelines.

The tibionavicular ligament

The TNL has been described in many anatomical literature as a component of the superficial layer of the DLC [12,34-37] with its reported prevalence ranging widely from 0% [15] to 100% [10,14,16] in cadaveric studies and MRI studies from 55% [38,39] to 100% [40]. It was present in 100% of the specimens in our study. The discrepancy in its prevalence may be due to its thinness, the unclear direction of its fibers, and its fusion with the ankle joint capsule [41]. Our study shows that TNL was the longest ligament, consistent with Panchani et al. [19], with a mean length of 38.57mm, which in turn concurs with three other cadaveric studies of 35 ankles in a meta-analysis with a pooled mean of 38.5 mm [21]. This suggests that the TNL anatomy in the Southeast Asian population is comparable to the Caucasian population.

The tibiocalcaneal ligament

The prevalence of TCL has been reported to range from 75% to 100% [10,15,16,38,39] with one study revealing a low prevalence of 15% [14]. In the present study, TCL could be distinguished in all specimens with a mean length of 34.29 mm, slightly longer than the mean average of 29.7 mm as reported in a meta-analysis of European cadaveric studies [21]. This suggests that during TCL reconstruction in Southeast Asian patients, special consideration should be given to positioning the calcaneal insertion point approximately 4 mm to 5 mm more distally compared to Caucasian patients.

The deep posterior tibiotalar ligament

The posterior tibiotalar ligament (PTTL) has been described differently across studies, with anatomical studies dividing it into superficial and deep layers [10,14-16,20], while MRI and sonographic studies consider it a portion of the deep layer of the DLC [38-40,42]. The prevalence of dPTTL was reportedly 100% [10,14-16], similar to our study findings. Our study reports a mean length of 18.71 mm, which is slightly longer than that reported in a meta-analysis study of European cadaveric studies at 15 mm [21]; though similar, it remains the shortest band of the DLC. This suggests that during dPTTL reconstruction in Southeast Asian patients, special consideration should be given to positioning the talus insertion point approximately 3 mm more distally compared to Caucasian patients.

The deep anterior tibiotalar ligament

The anterior tibiotalar ligament (ATTL) has been described as a component of the deep layer of the DLC [14], with the prevalence of dATTL reported widely ranging from 10% to 93% across various cadaveric studies [10,14-16,38,40]. It was present in all specimens. Our study reported a significantly longer dATTL with a mean length of 20.29 mm compared to a meta-analysis study of European cadaveric studies with a reported mean length of 12.85 mm [21]. This suggests that during dATTL reconstruction in Southeast Asian patients, special consideration should be given to positioning the talus insertion point approximately 8 mm more distally compared to Caucasian patients. However, this length discrepancy should be interpreted with caution, as the dATTL location lies beneath the TNL and blends with the ankle joint capsule, making clear differentiation challenging, despite careful dissection attempts [21].

Limitations

To our knowledge, this is the first morphometric analysis of the DLC in the Southeast Asian population. However, we acknowledge that there are a few limitations to our study. First, only a small cohort of seven ankles was included in our study, and this may not be representative of the population variability in comparison to other cadaveric studies by Won et al., comprising 60 specimens [40] and Milner et al., comprising 40 specimens [14]. Second, while the specimens were screened for major degenerative changes or deformities and excluded if there was a history of ankle instability or surgical procedures, the average age of our cadaveric cohort is unknown and may not be representative of the younger or older Southeast Asia population. Further studies comprising a larger study cohort with the inclusion of demographic data of the specimens should be conducted to improve representation and generalizability. Third, the natural contiguous nature of the ligamentous bands of the DLC may have introduced some variability during their identification. In an attempt to avoid this limitation, the dissection process was done meticulously with each ligamentous band being independently identified and confirmed by two authors. Lastly, our study did not identify the sPTTL or TSL or measure the thickness of each ligamentous band, which may be indicative of its degree of contribution to ankle stability.

## Conclusions

The current study offers valuable insights into the morphology and anatomical variations of the DLC within the Southeast Asian population. By examining the structural characteristics of each component of the DLC, this research contributes to a more detailed understanding of its variability in this specific population. Understanding these variations is crucial as anatomical differences can significantly impact clinical approaches in surgery, rehabilitation, and diagnostic imaging. The findings presented in this study enhance our comprehension of the DLC’s role in joint stability, highlighting potential differences in structure and function that may not be evident in other populations. These findings also pave the way for further research into anatomical diversity, which can influence clinical protocols and techniques.

Moreover, the detailed understanding of the DLC's anatomy, as demonstrated by this study, holds significant potential for improving surgical interventions, particularly in anatomical repair and reconstruction procedures. By identifying the unique anatomical features and variations of the DLC in the Southeast Asian population, surgeons can develop more tailored and effective treatment plans for individuals within this demographic. This knowledge may lead to advancements in the precision of surgical procedures, reducing the risk of complications and improving recovery outcomes.

## References

[1] Earll M, Wayne J, Brodrick C, Vokshoor A, Adelaar R (1996). Contribution of the deltoid ligament to ankle joint contact characteristics: a cadaver study. Foot Ankle Int.

[2] Harper MC (1987). Deltoid ligament: an anatomical evaluation of function. Foot Ankle.

[3] Renstrom PA (1994). Persistently painful sprained ankle. J Am Acad Orthop Surg.

[4] Lin CF, Gross ML, Weinhold P (2006). Ankle syndesmosis injuries: anatomy, biomechanics, mechanism of injury, and clinical guidelines for diagnosis and intervention. J Orthop Sports Phys Ther.

[5] Koulouris G, Connell D, Schneider T, Edwards W (2003). Posterior tibiotalar ligament injury resulting in posteromedial impingement. Foot Ankle Int.

[6] Hintermann B, Valderrabano V, Boss A, Trouillier HH, Dick W (2004). Medial ankle instability: an exploratory, prospective study of fifty-two cases. Am J Sports Med.

[7] Schuberth JM, Collman DR, Rush SM, Ford LA (2004). Deltoid ligament integrity in lateral malleolar fractures: a comparative analysis of arthroscopic and radiographic assessments. J Foot Ankle Surg.

[8] Hintermann B (2003). Medial ankle instability. Foot and ankle clinics.

[9] Loozen L, Veljkovic A, Younger A (2023). Deltoid ligament injury and repair. J Orthop Surg (Hong Kong).

[10] Campbell KJ, Michalski MP, Wilson KJ, Goldsmith MT, Wijdicks CA, LaPrade RF, Clanton TO (2014). The ligament anatomy of the deltoid complex of the ankle: a qualitative and quantitative anatomical study. J Bone Joint Surg Am.

[11] Wenny R, Duscher D, Meytap E, Weninger P, Hirtler L (2015). Dimensions and attachments of the ankle ligaments: evaluation for ligament reconstruction. Anat Sci Int.

[12] Gray H (1995). Gray's Anatomy: The Anatomical Basis of Medicine and Surgery. https://books.google.com.sg/books/about/Gray_s_Anatomy.html?id=n8XyjgEACAAJ&redir_esc=y.

[13] Kelikian AS (2023). Anatomy of the Foot and Ankle: Descriptive, Topographic, Functional. https://www.google.com.sg/books/edition/Sarrafian_s_Anatomy_of_the_Foot_and_Ankl/_J-mEAAAQBAJ?hl=en&gbpv=1&dq=Sarrafian%27s+anatomy+of+the+foot+and+ankle:+descriptive,+topographic,+functional+2021&printsec=frontcover.

[14] Milner CE, Soames RW (1998). The medial collateral ligaments of the human ankle joint: anatomical variations. Foot Ankle Int.

[15] Boss AP, Hintermann B (2002). Anatomical study of the medial ankle ligament complex. Foot Ankle Int.

[16] Clanton TO, Williams BT, James EW (2015). Radiographic identification of the deltoid ligament complex of the medial ankle. Am J Sports Med.

[17] Cromeens BP, Kirchhoff CA, Patterson RM, Motley T, Stewart D, Fisher C, Reeves RE (2015). An attachment-based description of the medial collateral and spring ligament complexes. Foot Ankle Int.

[18] Muhle C, Frank LR, Rand T (1999). Collateral ligaments of the ankle: high-resolution MR imaging with a local gradient coil and anatomic correlation in cadavers. Radiographics.

[19] Panchani PN, Chappell TM, Moore GD (2014). Anatomic study of the deltoid ligament of the ankle. Foot Ankle Int.

[20] Pankovich AM, Shivaram MS (1979). Anatomical basis of variability in injuries of the medial malleolus and the deltoid ligament. II. Clinical studies. Acta Orthop Scand.

[21] Yammine K (2017). The morphology and prevalence of the deltoid complex ligament of the ankle. Foot Ankle Spec.

[22] Zhang L, Huang T, Li C (2024). Race and gender differences in anterior cruciate ligament femoral footprint location and orientation: a 3D-MRI study. Orthop Surg.

[23] Sepúlveda R, Capurro B, Moreno R (2012). Morphometric study and anatomical variations of the medial ligament of the talocrural joint. Int j morphol.

[24] Zeegers AV, van der Werken C (1989). Rupture of the deltoid ligament in ankle fractures: should it be repaired?. Injury.

[25] Haddad SL, Dedhia S, Ren Y, Rotstein J, Zhang LQ (2010). Deltoid ligament reconstruction: a novel technique with biomechanical analysis. Foot Ankle Int.

[26] Deland JT, de Asla RJ, Sung IH, Ernberg LA, Potter HG (2005). Posterior tibial tendon insufficiency: which ligaments are involved?. Foot Ankle Int.

[27] Ellis SJ, Williams BR, Wagshul AD, Pavlov H, Deland JT (2010). Deltoid ligament reconstruction with peroneus longus autograft in flatfoot deformity. Foot Ankle Int.

[28] Kitaoka HB, Luo ZP, An KN (1998). Reconstruction operations for acquired flatfoot: biomechanical evaluation. Foot Ankle Int.

[29] Haddad SL, Coetzee JC, Estok R, Fahrbach K, Banel D, Nalysnyk L (2007). Intermediate and long-term outcomes of total ankle arthroplasty and ankle arthrodesis. A systematic review of the literature. J Bone Joint Surg Am.

[30] Pisanu F, Ortu S, Corda M, Andreozzi M, Caggiari G, Manunta AF, Doria C (2021). Deltoid ligament reconstruction with autologous gracilis tendon in chronic medial ankle instability after ankle fracture surgery: a case report. Foot (Edinb).

[31] Higashiyama R, Sekiguchi H, Takata K, Endo T, Takamori Y, Takaso M (2020). Arthroscopic reconstruction of the anterior tibiotalar ligament using a free tendon graft. Arthrosc Tech.

[32] Whitlock KG, LaRose M, Barber H (2022). Deltoid ligament repair versus trans-syndesmotic fixation for bimalleolar equivalent ankle fractures. Injury.

[33] Xiao K, Xie W, An Y (2022). Anatomic repair of deltoid ligaments in acute injury with suture anchor technique. Orthopedics.

[34] Rosse C, Gaddum-Rosse P (1997). Hollinshead's Textbook of Anatomy. No Title.

[35] Paulsen F, Waschke J (2019). Sobotta Clinical Atlas of Human Anatomy Vol. 1. https://www.google.com.sg/books/edition/Sobotta_Clinical_Atlas_of_Human_Anatomy/t9CDDwAAQBAJ?hl=en&gbpv=1&dq=Sobotta+atlas+of+human+anatomy+I.&printsec=frontcover.

[36] Standring S (2008). Gray's Anatomy: The Anatomical Basis of Clinical Practice. American journal of neuroradiology.

[37] Agur AM, Dalley AF, Grant JCB (2013). Grant's Atlas of Anatomy. https://www.google.com.sg/books/edition/Grant_s_Atlas_of_Anatomy/SmflHHbed4EC?hl=en&gbpv=1&dq=Grant%27s+atlas+of+anatomy.+Lippincott+Williams+%26+Wilkins&printsec=frontcover.

[38] Mengiardi B, Pfirrmann CW, Vienne P, Hodler J, Zanetti M (2007). Medial collateral ligament complex of the ankle: MR appearance in asymptomatic subjects. Radiology.

[39] Perrich KD, Goodwin DW, Hecht PJ, Cheung Y (2009). Ankle ligaments on MRI: appearance of normal and injured ligaments. AJR Am J Roentgenol.

[40] Klein MA (1994). MR imaging of the ankle: normal and abnormal findings in the medial collateral ligament. AJR Am J Roentgenol.

[41] Won HJ, Koh IJ, Won HS (2016). Morphological variations of the deltoid ligament of the medial ankle. Clin Anat.

[42] Park JW, Lee SJ, Choo HJ, Kim SK, Gwak HC, Lee SM (2017). Ultrasonography of the ankle joint. Ultrasonography.

